# Structure–Property Relationship in Composite Superabsorbents: How Butyl Succinate Architecture Affects Water Uptake and Phytotoxicity?

**DOI:** 10.3390/gels12030227

**Published:** 2026-03-10

**Authors:** Maria S. Lavlinskaya, Maxim S. Kondratyev, Andrey V. Sorokin

**Affiliations:** 1 Biophysics and Biotechnology Department, Voronezh State University, 1 Universitetskaya Square, 394018 Voronezh, Russia; ma-ko@bk.ru (M.S.K.); andrew.v.sorokin@gmail.com (A.V.S.); 2 Laboratory of Cell and Molecular Biophysics, Sevastopol State University, 33 Universitetskaya Street, 299053 Sevastopol, Russia; 3 Laboratory of Structure and Dynamics of Biomolecular Systems, Institute of Cell Biophysics of the RAS, 3 Institutskaya Street, 142290 Pushchino Moscow Region, Russia

**Keywords:** composite superabsorbent, swelling, plasticization, phytotoxicity

## Abstract

Composite superabsorbents (C-SAPs) that combine synthetic and polysaccharide components hold great promise for sustainable agriculture. They improve water management and enable the controlled release of agrochemicals. However, increasing the polysaccharide content to enhance biodegradability often reduces water absorption capacity. In this study, we explore plasticization with succinic acid esters as a strategy to overcome this limitation. Our goal is to establish structure–property relationships between plasticizer architecture and C-SAP performance. A series of carboxymethyl cellulose-based superabsorbents was synthesized via radical copolymerization. They were then plasticized with 5 wt.% of dibutyl succinate, di-*sec*-butyl succinate, or di-*iso*-butyl succinate. The resulting materials were characterized using FTIR spectroscopy, differential scanning calorimetry, rheological tests, swelling kinetics, and phytotoxicity assays against oilseed radish and common oat. Increased plasticizer branching and molecular volume enhanced polymer network elasticity, lowered the glass transition temperature (by up to 6 °C), and increased the equilibrium swelling ratio by up to 64% compared to the unplasticized C-SAP (661 ± 17 vs. 402 ± 10 g/g). All plasticized C-SAPs retained more than 80% of their initial swelling capacity over five swelling–deswelling cycles across pH 3.0–9.2. They also showed no phytotoxicity at agriculturally relevant concentrations. These findings demonstrate that molecular engineering of plasticizer architecture enables simultaneous optimization of water absorption and environmental safety in C-SAPs for agricultural use.

## 1. Introduction

Composite superabsorbent polymers (C-SAPs) are hydrogels capable of absorbing and retaining water in amounts hundreds to thousands of times their own mass. These materials integrate both synthetic and polysaccharide links within their structure. Unlike fully synthetic analogues, which are used primarily in personal hygiene care [1], biodegradable composite superabsorbents are particularly promising for agricultural applications. Their use in this sector contributes to efficient water management, soil erosion control, and enhanced crop yields [2,3].

The primary advantage of employing C-SAPs in agriculture stems from biodegradable polysaccharide units, which facilitate polymer degradation by soil biota [4]. Beyond the ecological benefit—biodegradation enables more complete polymer utilization and reduces soil contamination—the breakdown of C-SAP by soil microorganisms also renders these materials suitable as platforms for next-generation slow-release fertilizers [5,6]. This feature is particularly critical for readily soluble nitrogen fertilizers, which are susceptible to leaching, and for hydrophobic pesticides that suffer from low bioavailability [7,8]. Adjusting the polysaccharide content in C-SAP allows for tunable biodegradation rates, thereby enabling controlled release of encapsulated fertilizers and pesticides [4,9,10]. Furthermore, C-SAP formulations can be tailored to specific crop types and soil conditions through the incorporation of pH-responsive polysaccharides such as chitosan or carboxymethyl cellulose [11,12,13,14]. In addition, the rational and targeted delivery of agrochemicals to the plant root zone reduces overall agrochemical usage, thereby lowering input costs for farmers and increasing the profitability of agricultural operations—with reported yield improvements of up to 90% [15].

However, numerous studies on the synthesis of C-SAP report a decrease in water-holding capacity with increasing polysaccharide content [16,17,18,19,20,21]. Thus, improving the environmental sustainability of the material comes at the expense of its primary function—namely, the ability to retain and release water.

A promising approach to overcoming this limitation is plasticization, which involves the incorporation of small molecules into the polymer matrix. These agents partially disrupt the internal structure by interfering with weak intermolecular interactions, thereby increasing chain mobility. This strategy is well established and widely employed in industrial polymer processing to facilitate shaping and to tailor physicochemical properties. Commonly used industrial plasticizers, such as dioctyl phthalate and dibutyl phthalate, are unsuitable for agricultural applications due to their high toxicity [22]. Nevertheless, the current scientific literature offers virtually no information on the plasticization of C-SAP or on methodologies for selecting appropriate plasticizers for this class of materials [23]. We identified only one report on the use of glycerol as a plasticizer for a superabsorbent based on soy protein isolate [24]. However, no significant effect of glycerol on water absorption was observed. In contrast, other studies have demonstrated the influence of plasticizers on the water absorption properties of Eudragit^®^ polyacrylate resins, where an appropriately selected plasticizer resulted in an almost twofold increase in this characteristic [25]. This gap highlights the critical need to identify non-toxic compounds capable of effectively modulating the water absorption properties of composite superabsorbent polymers.

We have previously demonstrated that the incorporation of 5 wt.% dibutyl succinate increases the equilibrium swelling ratio (ESR)—a quantitative parameter characterizing the water-holding capacity of SAPs—by disrupting hydrogen bonds formed between functional groups within the superabsorbent network, particularly among hydroxyl groups of polysaccharide fragments [20]. Succinic acid was selected as the basis for the plasticizer due to a combination of its relevant properties: its availability and its role in plant metabolism. Succinic acid functions as a plant signaling molecule and a growth stimulant [26]. To the best of our knowledge, no systematic investigations into the effects of succinic acid esters on the water absorption properties of composite superabsorbents have been conducted to date.

Accordingly, the aim of the present study is to establish structure–-property relationships between the molecular architecture of succinic acid esters derived from structurally diverse butyl alcohols and the physicochemical and phytotoxic properties of composite superabsorbents containing these esters as plasticizers.

## 2. Results and Discussion

### 2.1. Influence of Plasticizers on the Physicochemical Properties of Composite Superabsorbents

Composite superabsorbents were synthesized via radical aqueous solution copolymerization of partially neutralized acrylic acid with acrylamide and biodegradable sodium carboxymethyl cellulose (CMC; 20 wt.%) in the presence of *N*,*N*’-methylene-*bis*-acrylamide as a crosslinking agent and potassium persulfate as a thermal initiator. CMC was selected as the source of biodegradable moieties due to its availability, low cost, and water solubility. The comonomer and component ratios were chosen based on previous studies [21] to ensure maximum water absorption capacity. Succinic acid esters, namely dibutyl succinate (DBS), di-*sec*-butyl succinate (D*s*BS), and di-*iso*-butyl succinate (D*i*BS), were introduced as potential plasticizers into the CMC-based superabsorbent polymer (CMC-SAP) at a concentration of 5 wt.% to obtain CMC-SAP-5DBS, CMC-SAP-5D*s*BS, and CMC-SAP-5D*i*BS, respectively. The amount of plasticizer incorporated into the C-SAPs was determined on the basis of a previous study [21], which demonstrated that the addition of 5 wt.% provides the maximum increase in water uptake capacity.

**Figure 1:**
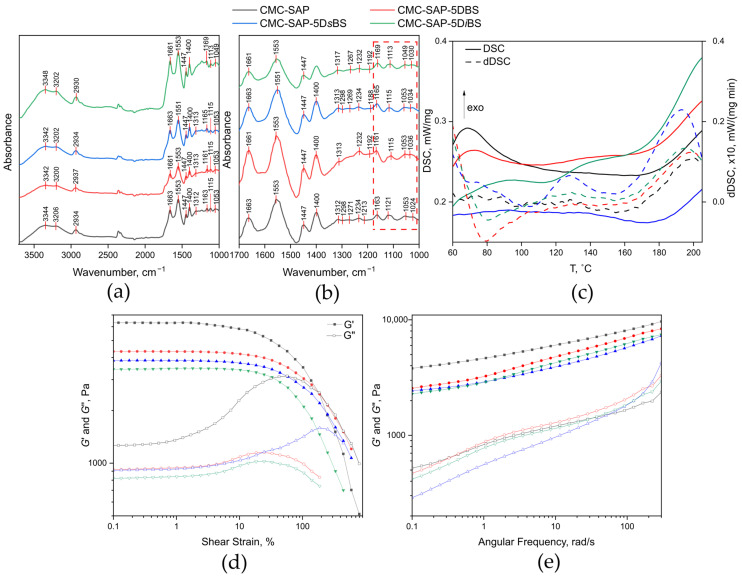
Physicochemical and rheological characterization of the obtained C-SAPs: (**a**) FTIR spectra in the 1000–3700 cm^−1^ range; (**b**) detailed FTIR spectra in the 1000–1700 cm^−1^ range, the red box marks the region corresponding to the vibrations of the pyranose rings and their associated OH groups; (**c**) DSC (solid lines) and dDSC (dashed lines) profiles; (**d**) storage (filled symbols) and loss (open symbols) moduli as a function of strain; (**e**) storage (filled symbols) and loss (open symbols) moduli as a function of frequency. The legend for all panels is shared above panels (**a**–**c**).

The structure of the obtained superabsorbents was confirmed by FTIR spectroscopy. Figure 1a shows a representative FTIR spectrum of the plasticizer-free CMC-SAP, which displays absorption bands confirming the incorporation of all components into the superabsorbent polymer. The bands in the 1024–1163 cm^−1^ region correspond to vibrations of the pyranose rings of CMC, C–O bonds (including C–OH vibrations of the pyranose rings), and glycosidic bonds [27]. A series of bands in the 1236–1312 cm^−1^ region, as well as those at 1447 and 2934 cm^−1^, are assigned to bending and stretching of C–H bonds [28]. The bands at 1400 and 1553 cm^−1^ arise from the symmetric and asymmetric stretching of carboxylate ions from acrylic acid and CMC [29]; the band at 1553 cm^−1^ overlaps with the amide II band (δ N–H and ν C–N) of acrylamide [30], which also appears as the amide I band (ν C=O) at 1663 cm^−1^ [31]. Stretching of OH and NH_2_ groups, along with water molecules associated with CMC-SAP, appear appears as a broad band with maxima at 3206 and 3344 cm^−1^ [32].

The FTIR spectra of the plasticized C-SAPs do not show additional absorption characteristic bands, which is likely due to the relatively low content of succinic acid esters (5 wt.%). However, shifts in the wavenumbers of several absorption bands are observed. These shifts are most pronounced for the bands corresponding to ν C=O and ν COO^−^, the bands in the 1024–1163 cm^−1^ region (attributed to skeletal vibrations of polysaccharides and their C–OH bonds), and the band associated with stretching of hydrogen-bonded OH groups at ~3344 cm^−1^ (Figure 1a,b). This suggests that the incorporation of butyl succinates into CMC-SAP induces an internal reorganization of the H-bonding network, as the bands that shift correspond to functional groups involved in hydrogen bonding. Notably, changes in the band shapes corresponding to CMC vibrations are also observed. This may indicate that the polysaccharide fragments, initially tightly bound by intra- and intermolecular hydrogen bonds, undergo the most pronounced reorganization.

Reorganization of the polymer internal structure influences its physicochemical properties, such as the glass transition temperature (*T_g_*). To assess the effect of plasticizer incorporation on *T_g_*, DSC profiles of the synthesized copolymers were recorded in the temperature range of 60–210 °C (Figure 1c). By comparing the inflections and maxima observed in the DSC and dDSC profiles, the glass transition temperatures were found to lie within the range of 193–199 °C (Table 1). The introduction of all tested esters resulted in a decrease in *T_g_*, indicating a reduction in weak intermolecular interactions within the polymer network. Notably, the magnitude of this reduction increased in the order: DBS < Di*s*BS < D*i*BS.

Taken together, the FTIR spectroscopy and DSC data suggest that the incorporation of butyl succinates into the CMC-SAP matrix disrupts the intramolecular hydrogen bonds formed between the functional groups. These interactions primarily occur between the OH and COO^−^ groups of CMC, as well as the COO^−^, NH_2_, and C=O groups of the acrylic comonomers. This disruption reduces the rigidity of the polymer network, enhances its chain mobility, and increases the accessibility of sorption sites, thereby improving the water uptake capacity of the superabsorbent. Accordingly, based on the observed changes in glass transition temperature, the effectiveness of the studied butyl succinates as plasticizers for composite superabsorbents (in terms of enhancing water uptake capacity) is expected to follow the order: DBS < Di*s*BS < D*i*BS.

The equilibrium swelling ratio (ESR; *Q_e_*) in distilled water confirms the role of each ester as a plasticizer that enhances water absorption capacity (Table 1). The unplasticized CMC-SAP exhibits an ESR of 402 ± 10 g/g, whereas incorporating 5 wt.% DBS, D*s*BS, or D*i*BS into the superabsorbent increases *Q_e_* by 14%, 45%, and 64%, respectively. Notably, the ESR values of the superabsorbents synthesized in this study are comparable to, or exceed, those of commercially available products. For instance, Aquasorb^®^, Akvasin^®^, and Zeba^®^ exhibit ESR values of 474 ± 29, 459 ± 40, and 358 ± 17 g/g, respectively.

Given the growing interest in composite superabsorbents, their development has become an active area of research. Table 2 compares the key characteristics of composite superabsorbents with similar compositions analogous to those in this study. The ESR values achieved in this work are competitive with—and in some cases exceed—those of previously reported materials. These findings underscore the potential of the developed superabsorbents for agricultural applications.

**Figure 2:**
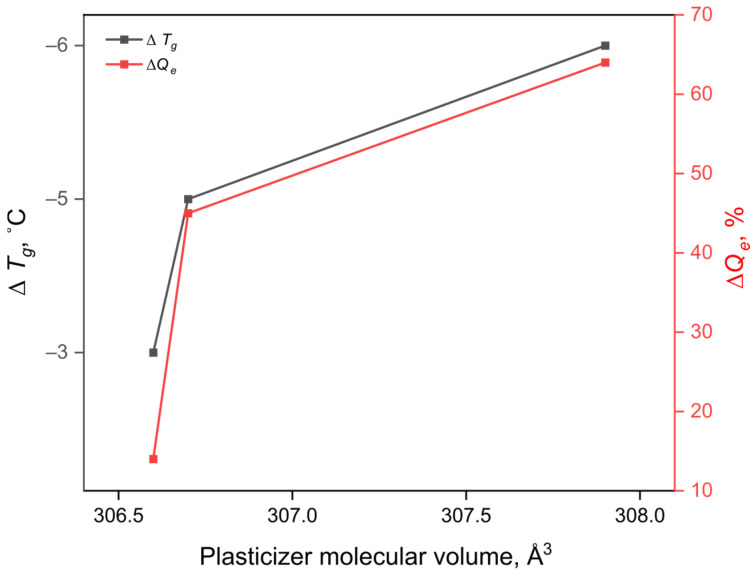
Correlation between plasticizer molecular volume and changes in glass transition temperature and ESR values.

The effectiveness of a plasticizer in modifying polymer properties is governed by its ability to disrupt weak intra- and intermolecular interactions, thereby increasing macromolecular mobility. This effect is often dependent on the plasticizer’s molecular volume and structure [40]. To interpret the observed trends, the molecular volumes of the succinic acid esters were calculated (Table 1). The data indicate that molecular volume increases in the order DBS < Di*s*BS < D*i*BS. This trend strongly correlates with the observed equilibrium swelling ratio and corresponding changes in the glass transition temperature of the obtained C-SAPs (Figure 2).

**Figure 3:**
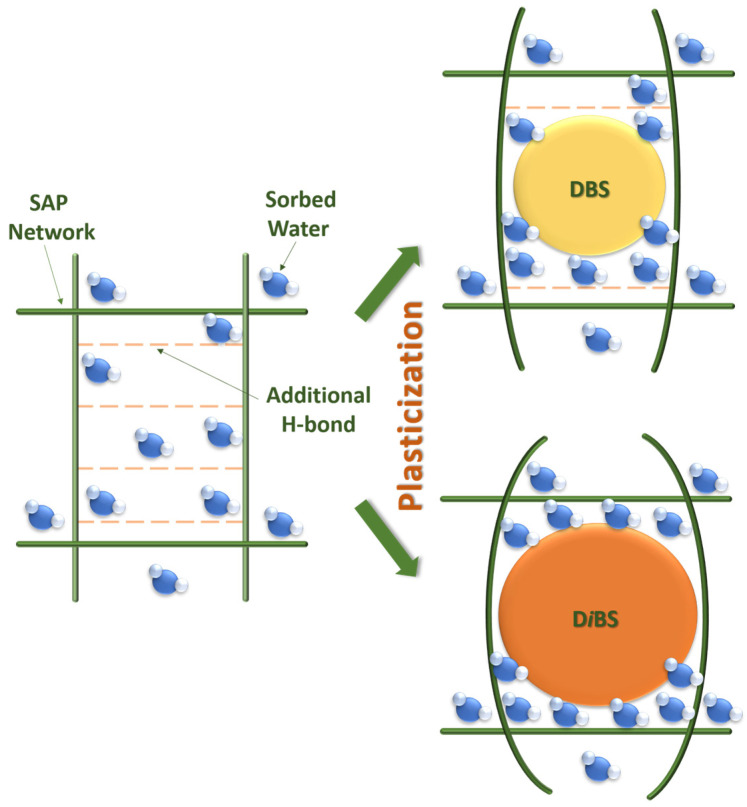
Schematic illustration of the interactions between different succinic acid esters and the C-SAP polymer network.

Accordingly, di-*iso*-butyl succinate, characterized by the most branched structure and largest molecular volume, blocks the formation of the greatest number of additional hydrogen bonds within the polymer network. This maximally reduces network rigidity and renders the largest number of sorption sites available for water interaction. In contrast, dibutyl succinate and di-*sec*-butyl succinate possess smaller molecular volumes and consequently block fewer additional hydrogen bonds, diminishing their efficacy as plasticizers for enhancing water absorption capacity (Figure 3).

In the soil environment, superabsorbents are subjected to continuous static and dynamic stress from soil particles. Composite superabsorbents are known to effectively retain their viscoelastic properties under such conditions [41]. However, the incorporation of a plasticizer alters the polymer network structure by hindering the formation of crosslinking hydrogen bonds. This modification may compromise the stability of the final product. Consequently, it is essential to evaluate how the addition of a plasticizer affects the stability and elasticity of the resulting material.

Figure 1d shows the storage modulus (*G*′) and loss modulus (*G*″) as functions of shear strain for the synthesized superabsorbents. To facilitate comparison, all samples were tested under identical conditions at a swelling ratio of 90 times their dry weight. For CMC-SAP, the storage modulus consistently exceeds the loss modulus across the entire strain range. Such a relationship confirms the formation of a stable, robust three-dimensional network. It also points to dominant gel-like elastic behavior with efficient energy storage and recovery. The linear viscoelastic (LVE) region extends up to shear strain (γ) ≈ 3%. For the plasticized C-SAPs, the relationship *G*′ > *G*″ is retained; however, the values of the storage modulus are lower than those of CMC-SAP. Notably, the LVE region is somewhat broader, extending to γ < 3% for CMC-SAP-5D*i*BS and CMC-SAP-5D*s*BS, and up to γ ≈ 5% for CMC-SAP-5D*i*BS. Thus, at an equivalent swelling ratio, the plasticized C-SAPs exhibit reduced brittleness and can withstand higher deformations without structural failure, attributed to their softer and more mobile polymer network.

The strain sweep dependence of the storage and loss moduli (Figure 1e) is consistent with the frequency sweep results and confirms the structural stability of the plasticized C-SAPs. Across the entire frequency range studied, the elastic component predominates (*G*′ > *G*″), and the moduli exhibit a linear dependence on frequency without sharp inflections. The frequency-dependent viscoelastic behavior demonstrates the long-term stability of the superabsorbents under constant soil pressure. Overall, all studied superabsorbents exhibited comparable and stable *G*′ values. This ensures structural integrity under prolonged load, thereby preserving soil porosity for sustained agricultural efficiency.

To retain their water storage and release properties in soil, superabsorbents must exhibit an elastic modulus comparable to that of soil, which typically falls within the range of 10^3^–10^6^ Pa [42,43]. Although the plasticized C-SAPs show a slight decrease in *G*′ values compared to CMC-SAP, these values remain within the required range. Overall, rheological results suggest that the obtained C-SAPs will exhibit high resistance to sustained pressure from soil particles. Furthermore, they will withstand cyclic loads without mechanical degradation of the polymer network.

### 2.2. Effect of Plasticizers on the Swelling Performance of Composite Superabsorbents

**Figure 4:**
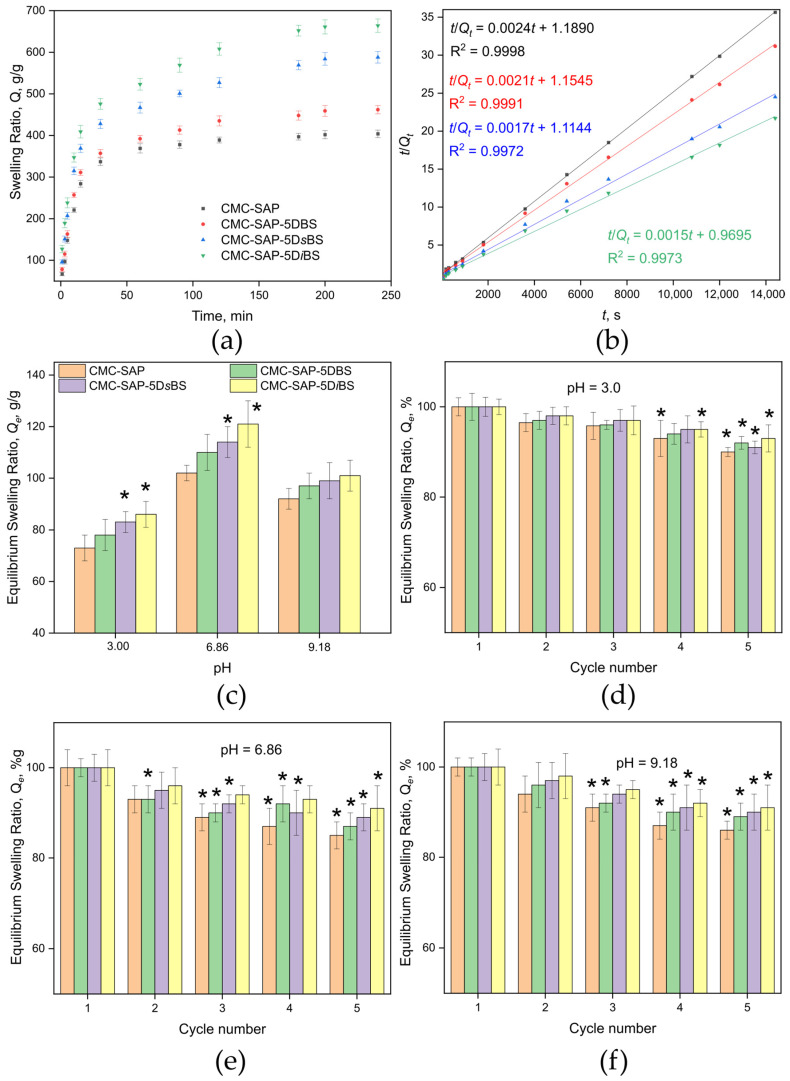
Evaluation of the swelling performance of the obtained C-SAPs: (**a**) swelling kinetic profiles; (**b**) swelling kinetic data processed with Schott’s pseudo-second-order model; (**c**) ESR values at different pH levels; (**d**) ESR dependence over five swelling–deswelling cycles at pH 3.00; (**e**) ESR dependence over five swelling–deswelling cycles at pH 6.86; (**f**) ESR dependence over five swelling–deswelling cycles at pH 9.18. The legend for panels (**a**,**b**) is shared in panel (**b**); for the remaining panels, the legend is provided in panel (**c**). Asterisks (*) indicate statistically significant differences (*p* < 0.05): for (**c**), CMC-SAP was used as the control; for (**d**–**f**), the values from the first cycle were used as the control.

Reorganization of the internal network structure of composite superabsorbents can influence the swelling kinetics. To investigate this, swelling kinetic profiles in distilled water were obtained for the composite superabsorbents (Figure 4a). The profiles clearly exhibit distinct regions of rapid (0–~50 min) and slow swelling (~50–~200 min), followed by an equilibrium plateau. All studied superabsorbents display similar profile shapes, characteristic of materials exhibiting non-Fickian diffusion during bulk processes [44]. Thus, the incorporation of plasticizers does not alter the general swelling behavior of the composite superabsorbents over time. However, the swelling curves for the plasticized C-SAPs show a steeper initial slope, suggesting a higher swelling rate during the early stages of the process compared to CMC-SAP.

To confirm this observation, the kinetic data were fitted using Schott’s pseudo-second-order model (Figure 4b), which enables determination of the equilibrium swelling ratio, initial swelling rate, and second-order swelling rate constant. The coefficient of determination (R^2^ > 0.99) obtained for all C-SAPs confirms the adequacy of the model for the experimental data. This further indicates that water sorption (superabsorbent hydration) occurs not only through interactions of water molecules with surface sorption sites of the C-SAPs but also within the bulk of the polymers [45].

The calculated equilibrium swelling ratios (*Q_e_^calc^*, Table 3) are in good agreement with the experimental data, confirming that the composite superabsorbents become fully saturated with water in less than 250 min. Notably, the initial swelling rate (*k_i_*) is higher for the plasticized samples compared to CMC-SAP and increases in the order CMC-SAP-5DBS < CMC-SAP-5D*s*BS < CMC-SAP-5D*i*BS, indicating the increasing availability of sorption sites for interaction with water. In this same series, a decrease in the swelling rate constant (*k_s_*) is observed. This constant characterizes the viscoelastic resistance of the polymer network to expansion during swelling. Thus, the swelling kinetics data correlate well with the DSC and rheological results, highlighting the increased elasticity and “softness” of the polymer network upon plasticizer incorporation, as reflected by the decrease in *T_g_* and *G*′ values, as well as the broadening of the LVE region.

Once introduced into the soil, superabsorbents are subjected not only to constant pressure from soil particles but also to the effects of pH and the ionic strength of the surrounding environment. Furthermore, due to periodically recurring cycles of rainfall and drought, superabsorbents undergo multiple swelling–deswelling events, which may affect their efficiency as water reservoirs. Therefore, the aim was to evaluate the effect of plasticizers on the swelling performance of the obtained C-SAPs within a pH range of 3.0–9.2, corresponding to the pH values of various soils [46]. Their behavior under repeated swelling–deswelling cycles was also assessed.

Figure 4c presents the ESR values of the C-SAPs in 0.1 M buffer solutions at pH 3.0, 6.86, and 9.18. The *Q_e_* values in these media are significantly lower than those in distilled water, an effect attributed to the presence of electrolytes in the buffer solutions, which suppress the dissociation of the polyelectrolyte C-SAPs. This suppression reduces electrostatic repulsion within the polymer network, leading to a substantial decrease in superabsorbent swelling. The effect is most pronounced under acidic conditions (pH 3.0), where the dissociation of unionized carboxyl groups from acrylic acid residues is also inhibited (the C-SAPs were synthesized using acrylic acid neutralized to 70 mol%). Overall, this behavior is consistent with the expected and characteristic response of polyelectrolyte hydrogels [47].

The behavior of the synthesized C-SAPs during swelling–deswelling cycles (Figure 4d–f) correlates with the rheological results. It confirms the formation of a stable, elastic polymer network that retains more than 80% of its initial ESR value over five cycles, regardless of pH. The incorporation of plasticizers enhances network elasticity, leading to a tendency for greater ESR retention in the plasticized samples. Furthermore, the preservation of high ESR values after five swelling–deswelling cycles indirectly indicates that the plasticizer is not leached out over the course of the experiment. This is supported by the lower tendency for ESR to decrease in plasticized C-SAPs compared to the unplasticized CMC-SAP. Overall, the swelling performance under varying pH conditions and cyclic swelling–deswelling demonstrates the potential of the developed materials for agricultural applications across a wide range of soil types.

### 2.3. Phytotoxicity Assay

Although plasticizers are known to improve the elasticity and durability of polymer networks, their incorporation into superabsorbents for agricultural use remains largely unexplored. To date, no studies have addressed the synthesis or application of plasticized C-SAPs, leaving a critical gap in understanding their environmental safety. Therefore, evaluating the phytotoxicity of such materials is essential to assess their potential impact on soil health and plant growth prior to agricultural deployment.

The concentration range of superabsorbents for phytotoxicity testing (0.25–3.00 mg/cm^2^) was selected based on the review by Zheng et al. [48], which indicates that the most effective agricultural application of composite superabsorbents corresponds to a rate of 45 kg/ha, or ~0.45 mg/cm^2^.

**Figure 5:**
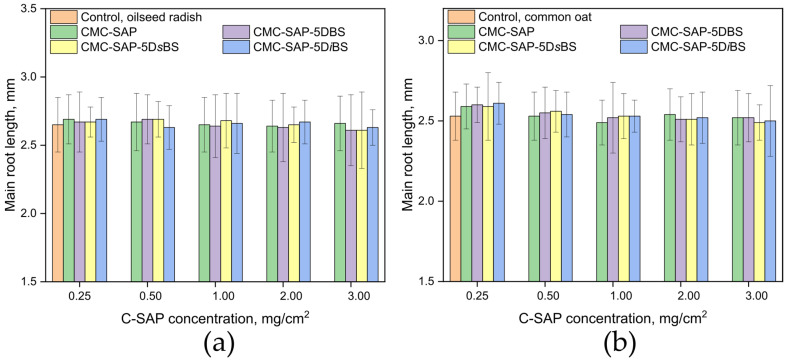
Root length of oilseed radish (**a**) and common oat (**b**) as a function of C-SAP concentration. No statistically significant difference from the control was observed for any of the C-SAP concentrations tested (*p* > 0.05).

The phytotoxicity results for the obtained C-SAPs are presented in Figure 5. All investigated superabsorbent formulations exhibited no inhibitory effect on the seed germination of the dicotyledonous oilseed radish (*Brassica rapa*) or the monocotyledonous common oat (*Avena sativa*) within the concentration range of 0.25–3.00 mg/cm^2^ (25–300 kg/ha). Thus, all obtained composite superabsorbents, including those plasticized C-SAPs with 5 wt.% dibutyl succinates of various structures are non-phytotoxic toward oilseed radish and common oat seeds at the tested concentrations.

Furthermore, these phytotoxicity findings provide indirect evidence that prolonged contact with the superabsorbents does not result in the leaching of toxic substances at concentrations hazardous to plants. This, in turn, confirms both the high conversion of the monomers used in synthesis and the effective purification of the final product from residual monomers. These factors are important not only for the efficiency and cost-effectiveness of raw material utilization in superabsorbent synthesis but also for reducing the anthropogenic impact on agricultural soils.

## 3. Conclusions

This study demonstrates that the incorporation of succinic acid esters as plasticizers effectively addresses the fundamental trade-off between biodegradability and water absorption capacity in polysaccharide-based composite superabsorbents. The molecular architecture of the plasticizer critically determines its efficacy: increasing branching and molecular volume from dibutyl succinate to di-*iso*-butyl succinate progressively enhances polymer network elasticity, reduces glass transition temperature, and increases equilibrium swelling ratio by up to 64% relative to unplasticized CMC-SAP. The plasticized C-SAPs retain stable swelling performance across agronomically relevant pH conditions and multiple swelling–deswelling cycles, while phytotoxicity assays confirm their safety for agricultural deployment. These findings establish a rational framework for plasticizer selection in C-SAP design, demonstrating that succinate esters can simultaneously improve water absorption and maintain environmental compatibility.

A limitation of this study is that plasticizer selection remains empirically driven and relies on extensive wet chemistry experimentation. This approach slows the accumulation of experimental data and does not yet sufficiently advance the theoretical understanding of plasticizer action mechanisms in superabsorbent polymers. Therefore, future research should focus on developing efficient and accessible approaches for predicting suitable plasticizers for C-SAPs using computational tools such as Hansen Solubility Parameters or molecular dynamics simulations. This should be complemented by experimental studies on the behavior of plasticized composite superabsorbents in soil, including plasticizer leaching, changes in ESR values after soil contact, and biodegradation.

## 4. Materials and Methods

### 4.1. Materials

The composite superabsorbent polymers were synthesized using sodium carboxymethyl cellulose (molecular weight 90 kDa, degree of substitution 0.7), acrylamide (AAm; >98%), acrylic acid (AA; >98%), *N*,*N*’-methylenebisacrylamide (MBAAm; >98%), potassium persulfate (PPS; >98%), and potassium hydroxide (>98%), all supplied by Acros Organics (Geel, Belgium). For the preparation of plasticizers, succinic acid, along with *n*-butyl, *sec*-butyl, and *iso*-butyl alcohols, as well as *p*-toluenesulfonic acid (all of analytical grade), were sourced from Vekton (Saint Petersburg, Russia). Solvents including toluene and ethanol (analytical grade) were obtained from ReaKhim (Moscow, Russia), and distilled water (18.2 MΩ·cm, pH 6.7 ± 0.2) was used throughout. Buffer solutions at pH 3.0 (0.1 M citrate buffer), pH 6.86 (0.1 M phosphate buffer), and pH 9.18 (0.1 M borate buffer) were prepared following established procedures.

Prior to use, acrylic acid was purified by distillation under reduced pressure (boiling point 45 °C/15 mm Hg) and subsequently neutralized to 70 mol% with potassium hydroxide. Acrylamide and MBAAm were recrystallized from ethyl acetate, while PPS was recrystallized from deionized water. All other reagents were employed without additional purification.

Three commercially available superabsorbent polymers marketed in the Russian Federation were used as reference materials: Akvasin^®^ (Singer, Kazan, Russia), Aquasorb^®^ (SNF Group, Andrézieux, France), and Zeba^®^ (UPL Corporation, Mumbai, India).

### 4.2. Synthesis of Plasticizers

Succinic acid (2 mmol), the corresponding butyl alcohol (16 mmol), and *p*-toluenesulfonic acid (0.2 mmol) were placed in a thermostated reactor equipped with a reflux condenser and a Dean–Stark trap filled with toluene. The boiling mixture was maintained until water evolution ceased, after which the excess alcohol was removed by distillation. The crude products were purified by vacuum distillation at 125–126 °C/4 mmHg (dibutyl succinate), 114–117 °C/4 mmHg (di-*iso*-butyl succinate), and 110–113 °C/4 mmHg (di-*sec*-butyl succinate). The product yields ranged from 74% to 83%, and purities exceeded 96%, as determined by LCMS using an Agilent 1269 Infinity liquid chromatograph coupled with an Agilent 6230 TOF LC/MS high-resolution time-of-flight mass spectrometer (Agilent Technologies, Santa Clara, CA, USA).

### 4.3. Plasticizer Molecular Volume Calculation

To calculate the molecular volumes of dibutyl succinate, di-*sec*-butyl succinate, and di-*iso*-butyl succinate, their molecular structures were constructed using HyperChem (http://www.hypercubeusa.com/, accessed on 20 February 2026) and subsequently optimized via quantum chemical calculations employing the semi-empirical PM7 (Parametric Method 7) method. Molecular volume calculations were then carried out using the MOPAC 2016 software package (http://openmopac.net/, accessed on 20 February 2026).

### 4.4. Superabsorbent Synthesis

CMC (0.25 g) was dissolved in 7.5 mL of distilled water under vigorous stirring in a thermostated reactor equipped with a reflux condenser, mechanical stirrer, and nitrogen inlet for degassing. An aqueous solution (2.5 mL) containing AAm (0.25 g), AA (0.8 g, 70 mol% neutralized with KOH), MBAAm (0.003 g), and PPS (0.015 g) was then added to the reactor. The polymerization mixture was maintained at 80 °C for 2 h.

The resulting copolymer gel was ground, immersed overnight in ethanol to remove soluble impurities, and dried under vacuum to constant weight. To obtain plasticized C-SAPs, the ethanol-soaked gel was milled together with 0.0625 g of plasticizer dissolved in 5 mL of ethanol prior to the final drying step. The product yield after drying ranged from 87% to 93%.

### 4.5. Superabsorbent Instrumental Characterization

#### 4.5.1. Attenuated Total Reflectance Fourier-Transform Infrared Spectroscopy

The chemical structures of the synthesized C-SAPs were analyzed via Fourier-transform infrared spectroscopy in attenuated total reflectance mode (FTIR ATR). A Bruker Vertex 70 spectrometer (Bruker Corporation, Billerica, MA, USA), fitted with a diamond-based single-reflection ATR module, was used for spectral acquisition. Each sample was scanned 32 times across a range of 1000–4000 cm^−1^, and the procedure was repeated over four measurement cycles. The analysis was carried out on finely powdered samples.

#### 4.5.2. Differential Scanning Calorimetry

The glass transition temperatures (*T_g_*) of the C-SAPs were determined by differential scanning calorimetry (DSC) using a Netzsch STA 449 F3 Jupiter simultaneous thermal analyzer (NETZSCH Group, Selb, Germany). Measurements were performed under a helium atmosphere with sample masses of approximately 7 mg. The samples were placed in sealed aluminum pans and heated from 60 °C to 210 °C at a heating rate of 10 °C/min.

#### 4.5.3. Rheological Studies

Rheological measurements of the C-SAPs were performed on an MCR102 rotational rheometer (Anton Paar, Graz, Austria) equipped with a parallel-plate geometry (25 mm diameter plates, 1 mm gap). Temperature control was achieved using the lower heating unit and an active hood, both regulated by Peltier elements (P-PTD200, Anton Paar, Graz, Austria), ensuring temperature stability within ±0.1 °C. The viscoelastic behavior of the samples was assessed under oscillatory shear conditions at a constant temperature of 25 °C. Two types of measurements were conducted: amplitude sweeps over a strain range of γ = 0.1–300% at a fixed angular frequency of ω = 6.28 rad/s, and frequency sweeps over ω = 0.1–300 rad/s at a constant strain of γ = 0.1%.

### 4.6. Superabsorbent Swelling Performance Assay

The equilibrium swelling ratio and swelling kinetics of the synthesized C-SAPs in distilled water and buffer solutions were evaluated following the procedure reported in [21]. Briefly, dry C-SAP samples (0.2000 ± 0.0002 g) were immersed in either 500 mL of distilled water or 100 mL of the appropriate buffer solution (0.1 M citrate buffer, pH 3.0; 0.1 M phosphate buffer, pH 6.86; or 0.1 M borate buffer, pH 9.18) for 24 h to attain equilibrium swelling. The swollen C-SAPs were subsequently collected by filtration, gently blotted with filter paper to remove surface water, and weighed.

For swelling–deswelling experiments, equilibrium-swollen C-SAP samples were placed on a nonwoven support and dried to constant mass using an A&D MX-50 moisture analyzer (A&D Company, Tokyo, Japan). The dried samples were then re-immersed in the corresponding buffer solution until swelling equilibrium was re-established.

The equilibrium swelling ratio (*Q_e_*, g/g) and the swelling ratio at time *t* (*Q_t_*, g/g) were calculated using the following equation:

(1) $$Q=\;\frac{m_{1}-\;m_{0}}{m_{0}},$$

where *m*_1_ and *m*_0_ are the weights of the swollen and dry C-SAPs (g), respectively.

The swelling kinetics of the synthesized C-SAPs were analyzed using Schott’s pseudo-second-order model [49]:

(2) $$\frac{t}{Q_{t}}=\;\frac{1}{k_{s}Q_{e}^{2}}+\;\frac{t}{Q_{e}},$$

where *k_s_* (g/(g sec)) is swelling rate constant. The initial swelling rate *k_i_* (g/g·s) as *t*→0 can be derived from the model parameters as *k_i_* = *k_s_Q_e_*^2^. Substituting this relationship into Equation (2) yields:

(3) $$\frac{t}{Q_{t}}=\;\frac{1}{k_{i}}+\;\frac{t}{Q_{e}}$$

By plotting *t/Q_t_* against *t*, a linear relationship is obtained. The kinetic parameters are then determined from the plot: *Q_e_* is calculated as the reciprocal of the slope, *k_i_* corresponds to the intercept with the ordinate axis, and *k_s_* is derived from these values using the relationship *k_s_* = *k_i_*/*Q_e_*^2^.

For each synthesized C-SAP, three replicate samples were examined (*n* = 3). The data are expressed as means ± standard deviation, calculated from three independent measurements using MS Excel 2019 software. Statistical analysis for swelling–deswelling data was conducted using Dunnett’s test for multiple comparisons against the control (cycle 1; *p* < 0.05).

### 4.7. Phytotoxicity Assay

The phytotoxicity of the synthesized C-SAPs was evaluated using seeds of a dicotyledonous plant, oilseed radish (*Brassica rapa*), and a monocotyledonous plant, common oat (*Avena sativa*). The assessment was based on the mean primary root length of seedlings measured over three days of incubation following root emergence at room temperature. For each test, 15 visually intact seeds were placed in a Petri dish (*d* = 10 cm) lined with filter paper, followed by the addition of 20 mL of an aqueous C-SAP dispersion at concentrations ranging from 0.25 to 3.00 mg/cm^2^, ensuring that the seeds were half-immersed in the liquid. A Petri dish containing 20 mL of water served as the control.

All measurements were performed in triplicate, and the results are reported as the mean ± standard deviation (*n* = 3). Statistical analysis was performed using one-way ANOVA, followed by Tukey’s HSD post hoc test for multiple comparisons within each concentration (*p* > 0.05).

## Figures and Tables

**Table 1:** Characteristics of the synthesized superabsorbents and plasticizers.

SAP	$T_{g}$, °C	Δ$T_{g}$, °C	$Q_{e}$ *, g/g	Δ$Q_{e}$, %	Plasticizer Molecular Volume, Å^3^
CMC-SAP	199	-	402 ± 10	-	-
CMC-SAP-5DBS	196	–3	459 ± 13	14	306.6
CMC-SAP-5D*s*BS	194	–5	584 ± 15	45	306.7
CMC-SAP-5D*i*BS	193	–6	661 ± 17	64	307.9

* *Q_e_* values determined in distilled water.

**Table 2:** Comparison of ESR values and application fields of the obtained C-SAPs with those previously reported.

C-SAP Composition	Initiation	$Q_{e}$ *, g/g	Application	Ref.
CMC, acrylamide (AAm), 2-acrylamido-2-methylpropanesulfonic acid (AMPS), *N*,*N*′-methylene-*bis*-acrylamide (MBAAm)	Ammonium persulfate (APS)	313	Soil conditioner	[18]
CMC, AA, MBAAm	Potassium persulfate (PPS)	545	-	[29]
CMC, acrylic acid (AA), MBAAm, LM EGaIn (75 wt.% Ga and 25 wt.% In)	APS	1108	Hygienic products	[33]
CMC, AMPS, MBAAm	PPS	178.7	Soil conditioner	[34]
CMC, AA	γ-irradiation	437	Hygienic products	[35]
CMC, AA, AAm, MBAAm, carclazyte	APS	515	Slow-release fertilizer/water management/drug delivery	[36]
CMC, AA, AAm, MBAAm, ammonium carbonate	PPS	1435	-	[37]
CMC, AAm, MBAAm, montmorillonite	PPS	721	Slow-release fertilizer	[38]
CMC, AAm, MBAAm,	APS	158	-	[39]
CMC, AA, AAm, MBAAm, di-*iso*-butyl succinate	PPS	661	Soil conditioner	This study

* Values determined in distilled water.

**Table 3:** Swelling kinetic parameters obtained from Schott’s pseudo-second-order model.

SAP	$Q_{e}$ *, g/g	${Q_{e}}^{\mathrm{calc}}$ *, g/g	$k_{i}$, g/(g s)	$k_{s}$ 10^6^, g/(g s)
CMC-SAP	402 ± 10	417	0.841	4.990
CMC-SAP-5DBS	459 ± 13	476	0.866	3.709
CMC-SAP-5D*s*BS	584 ± 15	588	0.897	2.593
CMC-SAP-5D*i*BS	661 ± 17	667	1.032	2.321

* Values determined in distilled water.

## Data Availability

The original contributions presented in the study are included in the article/Supplementary Materials, and further inquiries can be directed to the corresponding author.

## Supplementary Materials

The following supporting information can be downloaded at: https://www.mdpi.com/article/10.3390/gels12030227/s1, Raw FTIR, DSC and rheological data.
